# The role of the dipole moment orientations in the crystallization tendency of the van der Waals liquids – molecular dynamics simulations

**DOI:** 10.1038/s41598-019-57158-4

**Published:** 2020-01-14

**Authors:** Kajetan Koperwas, Karolina Adrjanowicz, Andrzej Grzybowski, Marian Paluch

**Affiliations:** 1 University of Silesia in Katowice, Institute of Physics, 75 Pułku Piechoty 1, 41-500 Chorzów, Poland; 2 Silesian Center for Education and Interdisciplinary Research SMCEBI, 75 Pułku Piechoty 1a, 41-500 Chorzów, Poland

**Keywords:** Chemical physics, Condensed-matter physics, Phase transitions and critical phenomena

## Abstract

Computer simulations of model systems play a remarkable role in the contemporary studies of structural, dynamic and thermodynamic properties of supercooled liquids. However, the commonly employed model systems, i.e., simple-liquids, do not reflect the internal features of the real molecules, e.g., structural anisotropy and spatial distribution of charges, which might be crucial for the behavior of real materials. In this paper, we use the new model molecules of simple but anisotropic structure, to studies the effect of dipole moment orientation on the crystallization tendency. Our results indicate that proper orientation of the dipole moment could totally change the stability behavior of the system. Consequently, the exchange of a single atom within the molecule causing the change of dipole moment orientation might be crucial for controlling the crystallization tendency. Moreover, employing the classical nucleation theory, we explain the reason for this behavior.

## Introduction

Cooling of any liquid results in its solidification. Using this protocol two different solid can be produced. The liquid may form the crystal, which is characterized by long-range order of the atomic structure. However, if the change of temperature is fast enough, the crystallization can be omitted, and after cooling to the glass transition temperature, supercooled liquid transforms into the amorphous solid, which exhibits lack of the atomic order. A discussed possibility of the occurrence of two mentioned and completely different scenarios is one of the main reasons for the actual researches’ fascination with the solidification. It is due to that the both mentioned process are inherently related phenomena because appropriate suppression of the crystallization during cooling is a way to obtain the supercooled liquid and subsequently the amorphous solid. Therefore the determination of the physical quantities which favor or impede the crystallization is recognized as one of the most critical tasks for the contemporary condensed matter physics, which furthermore is expected to provide many industrial benefits^1–5^.

Intuitively, the reasons for different crystallization behavior of the molecular liquids are looking for within intermolecular interactions similarly as they affect numerous of the structural and dynamic features of the viscous liquids^6–8^. Therefore the computational techniques, which enable precise parameterization of intermolecular interactions, seems to be ideal method to study the crystallization process on the most fundamental level. Consequently, the molecular dynamics simulations of so-called simple-liquids (i.e., systems composed of many particles interacting via radially symmetric pair potential) have been commonly employed. The scientific interest in the simple-liquids is additionally justified because of the fact that the form of one of the most popular pair-wise potentials, i.e., Lennard Jones (LJ) potential, is theoretically derived on the base of interaction occurring between real molecules. The repulsive interaction is caused by the overlapping of the electron clouds at short distances, whereas the attractive interactions result from electrostatic interactions between permanent as well as induced dipole moments^9^. Therefore, the use of the LJ potential enables rational modeling of the physical interactions between molecules of real van der Waals liquids. Hence the simulations of simple-liquids interacting via LJ potential are commonly used alternative to complex and time-consuming simulations of all-atom, which are too elaborate and often impractical for fundamental studies on physical process occurring in real materials.

Consequently, during the last decades, the much scientific effort has been put to examine the effect of changes in the pair-wise potential or the composition of the system on the glass transition and crystallization processes^6,7,10–15^. For example, it has been pointed out the LJ mixtures composed of two types of molecules are more prone to supercooling then the standard Kob-Andersen system^16^, after removing of interaction between identical types of particles^17^. Nevertheless, it has to be stressed that identifying the fundamental role of the intermolecular interactions on glass-formation in multicomponent mixtures is more complex because of tortuous phase diagrams or necessity to define the interactions between particles of the different types. Therefore, the simple molecular liquids containing the particles of the same kind and interacting in an isotropic manner seem to be a better choice. Consequently, the simulations of simple-liquids interacting via LJ potential have been used to the systematic investigation of the effect of attractive (or repulsive) forces on the tendency to the formulation of the crystallization tendency^18–21^. It is of significant meaning for the real materials because as we mentioned in the previous paragraph the intermolecular attraction is closely connected with the permanent or induced dipole moments. Hence proper parameterization of the LJ potential gives the possibility to reflect differences in the intermolecular interactions causing by the different values of the dipole moments or polarizability for various molecules^20,21^. Given this fact, it has to mention that from the above studies one can conclude that the increase in repulsion and/or decrease in attraction result in the higher ability to form crystalline structures.

However, the modesty of simple liquids implies that many fundamental properties of the real material are entirely ignored for those model systems. One of the most evident is the structural anisotropy. Consequently, through the last decades, various model systems, which can be devoted to the two main groups, have been proposed to study the effect of structural anisotropy on the dynamic and thermodynamic properties. The first group of model systems treats molecules as hard objects with a given shape, in which anisotropy results from short-range repulsive forces resulting from impenetrability of hard-cores^22^. Performed studies show that the molecular shape may be crucial in determining the phase behavior as a function of the density. For example, the phase diagram of the hard-dumbbells, i.e., two hard spheres connected by the rigid bond, depends on the elongation^23–26^. At low elongations, the liquid freezes into a close-packed plastic crystal where the centers of mass of the molecules form an ordered lattice, but they can rotate. The FCC plastic crystal is stable at low elongations although it becomes metastable concerning the HCP when elongation (and pressure) increases^26,27^. Additionally, a further increase in elongation results in the transformation of plastic crystal to the orientationally order solid. The complex phase transitions behavior has been also reported for hard rods, which exhibits five different phases depending on the density and the shape anisotropy estimate by the ratio of length and width^28^. Hence, the shape of hard molecules seems to be crucial for determining the phase diagram as a function of the density - the temperature enters the thermodynamics in an only trivial way for these model systems^29^. In contrast to hard molecules, the second group of model systems considers the molecular anisotropy by modeling of intermolecular interaction occurring between nonspherical molecules. This approach enables to include a short-range repulsion as well as a long-range attraction as a function of the distance between molecules and their mutual orientations. It should be noted that for this type of molecules temperature is an essential thermodynamic variable, which influences the physical properties. Consequently, numerous potential models have been proposed within which the most popular are Kihara potential^30^, the Gaussian overlap model^31^, and the Gay-Bern potential^32^. However, from the experimental point of view, the most natural is an obvious phenomenological description of the intermolecular interaction which includes all atom-atom interactions. In that way, the structure of real molecules is reflected, and then closer agreement with the experiments may be expected. Consequently, dynamical^33–37^ and structural^38–41^ properties of many model liquids have been extensively studied through the last decades, proving that these model systems provide fairly good description of slightly non-spherical molecules^42^. It has to be also mentioned about another significant advantage of the atom-atom (or site-site) model potential, i.e., about the possibility of including the spatial distribution of charges within the molecule^39^. Hence, similarly to simple-liquids, these type of model systems could be used to study the effect attractive intermolecular forces resulting from interactions between electric dipole (or quadrupole) moments on dynamics and thermodynamics.

However, it must be pointed out that, in contrast to simple-liquids, the structural anisotropy implies that orientation of the dipole moment can be precisely defined, i.e., parallel or perpendicular positions of dipole moment in respect to given molecular axis can be easily distinguished. Therefore, combining the pointed out the effect of the value of the dipole moment on the crystallization process with the noticed importance of the structural anisotropy the natural question about the role of the dipole moment orientation for crystallization behavior arises. Consequently, the aim of this paper is to improve understanding of the mechanism of the crystallization by examination of the effect of different dipole moment orientations on this process. Using the model molecules, we specify which orientation of the dipole moment is preferable for formation of the crystalline structure. Moreover, our results show that expected impediment in the crystallization, which is caused by more complex structure of particles, could be overcome by interactions occurring between properly oriented dipole moments. Finally, we show that this finding can be explained in the framework of the classical nucleation theory.

**Figure 1:**
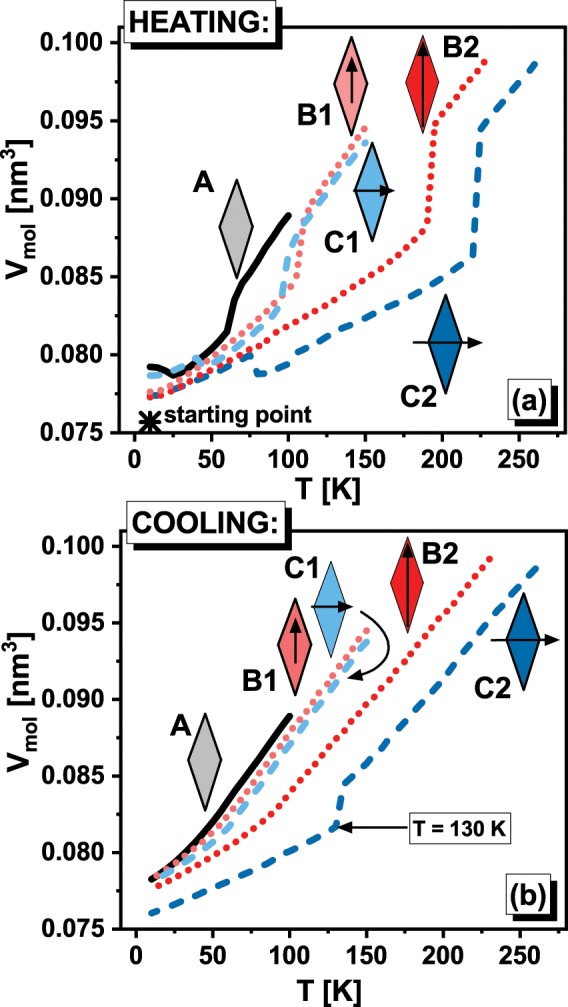
The temperature dependences of molecular volumes during heating (**a**) and cooling (**b**) for all studied systems are presented. The star denotes the initial FCC configuration, which was subsequently equilibrated at T = 10 K and p = 100 MPa. In panel (b) the temperature at which the crystallization takes place is indicated.

The investigations of the dipole moment orientation influence tendency to crystallization require the design of more complex systems than simple-liquids. It is due to the necessity of distinguishing between various orientations of the dipole moment, which requires favoritism of some molecular axis. However, to eliminate the effect of other factors on the considered process the designed particles should be as simple as possible. To meet the above conditions we created model molecules, which are comprised of four identical atoms (of carbon atom mass) arranged in a rhombus shape^43^. The shape of designed molecule implies that it possesses short and long molecular axis (along diagonals of rhombus) simultaneously keeping identical bonds lengths. We set the bond length to be equal to 0.14982 nm (0.14 nm is a bonds length for carbon atoms in benzene ring), whereas angles between bonds are established to make one diagonal two times longer than the other. The stiffness of bonds, angles and dihedrals, as well as the non-bonded interaction between atoms of different molecules, have been defined by OPLS all-atom force field^44^ parameters provided for carbon atoms of the benzene ring. To keep simplicity of constructed model molecules as much as possible, we decided not to add hydrogen atoms. As a consequence, we obtained systems of rhombus-like molecules (RLM), which to some extent mirror the flat and asymmetric shape of the real molecules. Subsequently, we redefine the charges for appropriate atoms of RLM to ±0.125*e*, ±0.25*e*, or ±0.5*e* (*e* is an elementary charge) to create weak and strong *μ* oriented alongside the long and the short molecular axis. Finally, we obtained 5 different systems; the schemes of their constructions are presented in the Fig. 1. RLM of type A does not have a dipole moment, the RLM of type B possess the dipole moments oriented alongside the long molecular axis, whereas RLM of type C are characterized by the dipole moment of orientation along the short molecular axis. Moreover, molecules B1 and C1 are characterized by the same value of *μ*, which is two times smaller than *μ* for molecules B2 and C2.

Our investigation we began from the construction of perfect FCC lattice crystal, in which the 2048 RLM were inserted in the place of atoms. The standard simulations of the molecular dynamics have been performed by use of the GROMACS software^45,46^ at conditions of constant temperature and the pressure controlled by the Nose-Hoover thermostat^47–49^ and Martyna-Tuckerman-Tobias-Klein barostat^50,51^ (*p* = 100 MPa) respectively. Each simulation run lasts for a relatively long time, i.e, 1 bilion of time-steps (*dt* = 0.001 *ps*). The first half of simulation was devoted for equilibration of the system, whereas the volumetric and dynamic data have been collected for the last 500 millions of time-steps. Then, we heated (Δ*T* = 5 K) the systems at constant pressure from the starting temperature equals 10 K. At this point we would like to mention that the initial FCC structure were proposed by use due to computational reasons. It protects system to undergo significant changes of the volume during the first steps of simulations at 10 K. Additionally, as one can see in Fig. 1a the initial structure forces systems to maintain some crystal structure, which immediately results that during heating process we observe the step increase in the volume for each system, which indicates on the melting of the crystal structure, see Fig. 1a where the temperature dependence of the molecular volume *V*_*mol*_ = *V*/*N* ($N$ denotes number of molecules within the system of volume, $V$) is presented. The heating was stopped at the temperature at least $35\,{\rm{K}}$ higher than the temperature of the detected drop in the density. During this stage one can observed that after equilibration at starting temperature the systems without $\mu$, and with $\mu$ oriented alongside the short molecular axis, i.e., systems A, C1, and C2 tend to form the more preferable (and denser) crystal structure than those resulted from initial FCC structure, whereas system B1 and B2 does not exhibit any phase transition to the another crystalline form. At this point we would like to put readers attention on the fact that some structural changes may occur also during equilibration process at 10 K. This fact enables us to expect that close to temperature of observed step increase in the volume analyzed systems exhibit thermodynamically stable crystal structures. Of course, those structure probably are not perfect. However, simplicity of designed molecules let us suspect that unit cell of their crystal form would not be complex and therefore the number of defects in comparison to the number of crystal cells is tiny. Consequently, we expect that influence of defects is negligible and therefore for our further theoretical analysis we will base on crystal structures registered during heating. Moreover similarly to the previous results for simple-liquids^19–21^, increase in the dipole moment, which implies gain in the attraction between molecules, causes that crystal phase become more stable, i.e., the temperature at which melting of the crystal structure is observed, become higher. Therefore, on the base of presented studies, we can confirm that this effect is independent on the orientation of the dipole moment.

After heating of the examined systems we started the main stage of our experiment, which is the isobaric cooling up to the starting temperature (Δ*T* = 5 K), see Fig. 1b. Interestingly, during cooling, the only one system exhibits the step decrease in the volume, which suggests the occurrence of the crystallization event. It is observed for system (C2), which is characterized by strong $\mu$ oriented alongside the short molecular axis. At this point, we would like stress that in order to verify whether this finding is repeatable, we performed three times cooling of systems C2 and B2 (for which temperatures of observed melting is the most nearing to temperature of observed melting of system C2) and all experiments ended identically.

Considering the results of systems cooling, which are comprised of various RLM we would like to put readers attention on certain key observation. Since the system C2 possesses higher value of $\mu$ than system C1, it is suspected that system C2 is characterize by the stronger attraction between molecules, and then, according to results of simple-liquid simulations, its tendency to crystallization should be smaller^19–21^. However, system C2 exhibits the sudden drop in volume indicating on crystallization whereas such behavior is not observed for system C1. Therefore, the straightforward relation between the gain in strength in the intermolecular attractions, which is induced by the value of the dipole moment, and tendency of the system to the formation of the crystalline structure is not evident for the investigated herein systems. Hence, behavior of RLM is in contrast to the recent results for the simple-liquids. As a consequence, the validity of discussed straightforward relationship cannot be expected for the real materials in general. Furthermore, this outcome suggests that the simulations of model systems, which are characterized by more complex architecture than the simple-liquids, e.g., RLM, could be a method for systematic and detailed studies of given intramolecular feature effect on structural, dynamic, and thermodynamic properties of the system. In this context it is worth to noting that the simplicity of RLM gives promising hope for theoretical solutions, which are not accessible for real materials (in this context we encourage readers to acquaint with our very recent paper where RLM are used to solving the important problem of the density scaling^43^).

It is also worth noting that during cooling of systems A, B1, B2, and C1 the change in the slope of the temperature dependences of the volumes, which is a manifestation of the glass transition, is observed. It implies that RLM seem to be interesting candidates to study the glass transition as well, especially because the common one-component model systems usually easily crystallize.

**Figure 2:**
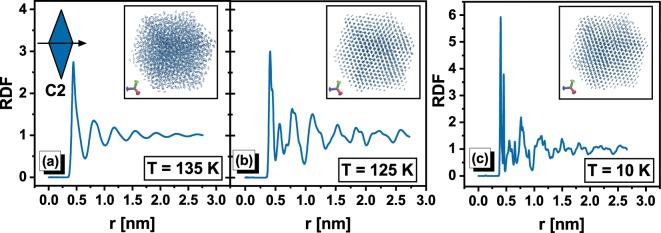
The radial distribution functions (RDF) are presented for system C2 at three different temperatures. In the insets, the snapshots of the simulation box at respective thermodynamic conditions are shown. Additionally, the scheme of RLM of system C2 is presented in Fig. 2a.

Additionally, it should be noted that system C2 likely exhibits the highest trend to crystallization independently on the pressure conditions because, especially for simple molecules, increase in the pressure facilitates the formation of the crystal form within the liquid. Thus, also at lower pressures exclusively system C2 is suspected to occur liquid-crystal phase transition. However, this key observation of our studies as well as aforementioned advantages of RLM could be recognized as valid only if the step decrease in the volume observed for system C2 in Fig. 1b is indeed a manifestation of the discussed process. Given this remark, we analyzed the structure of the system C2 at crucial thermodynamic conditions. Already at the first glance a comparison between the simulation box snapshots at T = 135 K (, which is just before the expected crystallization event, T = 130 K), T = 125 K, and at the final temperature (T = 10 K), reveals evident differences, see insets of Fig. 2a–c. RLM of system C2 at T = 125 K and at the final temperature are visibly arranged in manner characteristic for the crystal structure, whereas any similar arrangement is not observed at T = 135 K. At this thermodynamic condition, the structure of system C2 seems to be totally disordered (like in the case of liquid), which indicates on the process of rapid structure ordering. Above visual conclusion is in accord with the observed changes in the shape of the radial distribution function (RDF) calculated at the mentioned thermodynamic conditions. It is clearly seen in Fig. 2b,c that the shapes of RDF exhibit the distinctive for the crystal structure the sharp peaks. On the other hand, the molecular configuration at T = 135 K is characterized by RDF of much softer shape, which is typical for liquid structure. Moreover, the values of RDF at this temperature are about two times smaller than at the final temperature. Thus, the changes in RDF also confirm that observed in Fig. 1b steep decrease in the volume for system C2 is indeed the manifestation of the crystallization process.

**Figure 3:**
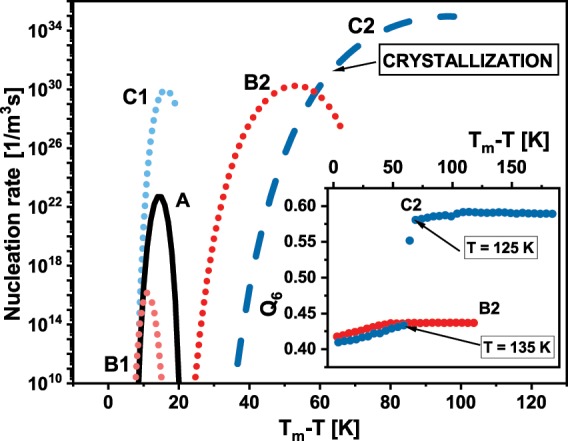
The nucleation rates for all studied system are presented. Additionally, the thermodynamic conditions of the crystallization process are marked. In the inset, the temperature dependences of the global bond-orientational order parameter for systems B2 and C2 are shown.

Another interesting point, which is worth to study, is the examination of the temperature behaviors of systems comprised of various RLM. However, the comparison between different shapes of RDF function is not the convenient and precise way for this analysis. Fortunately, it can be done using another standard tool for a characterization of the local/global structures which are the bond-orientational order parameters^52^. It is worth to note that these parameters are commonly used for identification of different crystalline phases^53–56^ as well as to study of the melting transitions^57–59^. For this purpose, i.e., for the studies of supercooled liquids and glasses, *q*_6_ and *Q*_6_ are considered as the most prominent parameters, the idea of which could be briefly described as follows. The local structure around particle *i* could be quantified by a set of numbers ${\bar{q}}_{lm}(i)\equiv \frac{1}{N(i)}\mathop{\sum }\limits_{j=1}^{N(i)}{Y}_{lm}({\hat{r}}_{ij})$, where *l* = 6 for *q*_6_, ${\hat{r}}_{ij}$ is a unit vector in the direction between particle *i* and one of its neighbours, *j*, which total number equals *N*(*i*), and ${Y}_{lm}({\hat{r}}_{ij})$ is a spherical harmonics. Then the average of ${\bar{Q}}_{lm}$ over all particles gives ${\bar{Q}}_{lm}$. Then its rotational invariant takes the following form ${Q}_{l}\equiv {(\frac{4\pi }{2l+1}\mathop{\sum }\limits_{m=-1}^{l}{|{\bar{Q}}_{lm}|}^{2})}^{1/2}$, which is finally recognized as a global order parameter (${Q}_{6}$ for $l=6$). The temperature dependence of the global bond-orientational order parameter, *Q*_6_, between the centers of the molecules is presented in the inset of Fig. 3 for the system C2 and B2. For this study, we defined the nearest neighbor as the particle which is not farther than 1/10 of the simulation box from the considered particle. One can see that during cooling *Q*_6_ increases in a consistent manner up to T = 135 K, i.e., up to the temperature below which the system C2 crystalizes. Then, *Q*_6_ rapidly rises, which means immediate ordering of the structure - crystallization. After this process the structure of the system remains practically unchanged. Interestingly any discontinuity (jump) in the value of *Q*_6_ is not detected for system B2. In this case *Q*_6_ increases up to T = 80 K and then remains constant. This behavior indicates freezing of the liquid structure. It is worth to mention that RDF for system B2 at the final temperature still possesses characteristic for liquid structure shape (results not presented). Interestingly the freezing of the structure takes place at the temperature for which characteristic for the glass transition change of the slope of the temperature dependence on volume, is observed.

Since from the five examined systems only one crystallizes, it is interesting to check whether the occurrence of crystallization process could be explained within the classical nucleation theory (CNT), which is the most widespread theoretical description of the discussed process. According to the CNT the crystallization is preceded by the formulation of the nuclei of critical size, which subsequently grows. Therefore, the nucleation rate of critical nucleuses, *J*, is the crucial quantity determining the occurrence of the crystallization event. In the term of CNT, the nucleation rate is expressed according to the following formula^60^,

1 $$J={\rho }_{l}^{4/3}\sqrt{\frac{\sigma }{{k}_{B}T}}D\exp (-\,\frac{\Delta W}{{k}_{B}T}),$$

where ${\rho }_{l}$ is the number density of the liquid, ${k}_{B}$ is the Boltzmann constant, $\sigma$ indicates solid-liquid interfacial free energy, *D* is a diffusion constant, and $\Delta W=\frac{16}{3}\frac{{\sigma }^{3}}{{(\Delta g)}^{2}}$ is the nucleation barrier ($\Delta g$ is a driving force for crystallization). Before the determination of the $\Delta g$, it is usefull to consider that for all examined systems we set identical isobaric condition, and therefore used pressure could define as the reference pressure. Then the driving force become a function of only temperature, and consequently the equation for driving force proposed by Gutzow^61^ with the redefined integration pathway^62^ takes the following form $\Delta g(T)=\,-\,{\int }_{{T}_{m}}^{T}\Delta S(T)dT$, where $\Delta S$ is the difference in the entropy between the liquid and the solid phases. $\Delta S$ was estimated using the obtained directly from the simulation-runs temperature dependence of the enthalpy, $H(T)$ and the classical relation between the enthalpy and the entropy, $T={(\partial H/\partial S)}_{p}$, (considering the melting boundary condition, $\Delta {S}_{m}=\Delta {H}_{m}/{T}_{m}$). At this point it has to be stressed that calculation of the $\Delta {S}_{m}$ and therefore also $\Delta g$ requires precise determination of the melting conditions. The latter can be done using method of the liquid-solid coexistence, which use the fact that at melting conditions the solid and liquid phases remain in the thermodynamic equilibrium. Hence the special biphasic simulation box consisting 4096 RLM divided equally between separated by a small gap crystal and liquid phases. The box was created by the connection of the crystal structure registered during heating and the liquid box of the same high and wide. The long of the liquid part of the biphasic box was set to ensure density of liquid corresponding to that registered during cooling process. Subsequently, we simulated the biphasic system at various temperatures (keeping constant pressure) and determined the temperature at which the coexistence of both phases is observed for 10 ns (1 billion time-steps). The distinction between crystal and liquid was possible even by visual examination. When the system exhibits coexistence of two phases for 10 ns we recognize given temperature as the melting temperature (if needed we extended simulation time to complete melting or crystallization of the biphasic system). The determined melting temperatures equal 50 K, 36 K, 128 K, 65 K, 194 K for system A, B1, B2, C1, C2 respectively. According to Eq. (1), calculation of the nucleation rate requires also determination of the solid-liquid interfacial energy, which was estimated from the formula suggested in ref. ^62^. At isobaric conditions of the reference pressure formula takes succeeding form $\sigma (T)={\sigma }_{0}-\frac{{g}_{0}}{{m}_{0}}{\int }_{{T}_{m}}^{T}\Delta S(T)dT$, where parameters ${g}_{0}$ and ${m}_{0}$ are defined in the way proposed by Gutzow and coworkers^61^. The value of ${\sigma }_{0}$ for each system was estimated using Scapski-Turnbull formula ${\sigma }_{0}={\gamma }_{0}\Delta {S}_{m}{T}_{m}/{m}_{0}$, where typical ${\gamma }_{0}\approx 0.4$ was employed. The last quantity necessary to calculation of $J$ is the diffusion constant, which was determined using GROMACS software from the means square displacement for long times. The obtained temperature dependence of $D$ was subsequently approximated by the Vogel-Fulcher-Tammann equation.

The curves of the nucleation rates are presented in Fig. 3 for all studied systems. It is seen that the nucleation rate is the highest for the system C2, i.e., for the system which indeed crystalizes. Additionally, one can observed that around the temperature at which crystallization occurs ($T=130\,K$, which is 64 K bellow the melting temperature) *J* for system C2 is 10 times higher than for C1 and B2 systems. This finding suggests that for other studied systems the nucleation rate is not sufficient for formation of the stable nuclei and then for the initiation of the crystallization process. Consequently, the crystallization does not take place for those systems. Moreover, we would like to note that the gain in the intermolecular attraction caused by increase in the dipole moment not always moves nucleation rate curves further from the melting temperature - maximum of *J* for system B1, which poses dipole moment (oriented alongside longer molecular axis), is closer to *T*_*m*_ than the maximum of *J* for system A, i.e., for the system without dipole moment. This fact puts new insight on the straightforward connection between value of the dipole moment, related to it intermolecular attraction, and the crystallization tendency, which have been achieved from the studies of simple-liquids^19–21^. For these systems increase in the intermolecular attraction results in the shift of the nucleation rate further from *T*_*m*_. Consequently, the separation between curves of nucleation and crystal growth rates become higher, which impedes occurrence of the crystallization process. However, in the case of studied herein systems, one can observe in Fig. 3 that the nucleation rate curves are closer to the melting temperature and then to the respective crystal grow rate curves (crystal growth rate, *U*, were calculated in the same way like in ref. ^20,21^, results not presented) for systems which do not crystalize. Consequently, the mismatch between nucleation and crystal grow, which is the highest for system C2 cannot be responsible for the occurrence of the crystallization process for the studied systems. On this base, one can suspect that the stability behavior of more complex systems than the simple liquids depends more significantly on the nucleation rate value than on the separation between *J* and *U* curves.

Moreover, comparing results obtained for all studied systems we can observe that playing the orientation and/or the value of the dipole moment it is possible to steer the nucleation rate. Creation of the dipole moment as well as the change of the existing one implies modification of the nucleation rate, see results for A, B1, and C1 presented in Fig. 3. However, it must be stressed that the obtainment of requested *J* behavior is a tricky task because molecular structure have to be considered as well. In should be also taken into account that too high the value of the dipole moment could lead to the overcoming of the previous decrease in *J* (caused by the given orientation of *μ*) and finally to the gain in nucleation rate (see results for B1 and B2 in Fig. 3).

## Conclusions

The simulations of the new model molecules, enable us to demonstrate that the interior orientation of the dipole moment has a significant role in the stability behavior of the liquid below the melting conditions. The change of the dipole moment arrangement could result in a total change of the tendency of the system to crystallization. Model molecules with the dipole moment oriented perpendicularly to their longest molecular axis exhibit higher tendency to the formulation of the crystal structure than those with the orientation of *μ*, which is parallel to the longest molecular axis. This result suggests that the impediment of the crystallization tendency, which is the effect of more complex molecular structure could be overcome by the interactions between properly arrange dipole moments. The determination of critical value of the dipole moment, which result in crystallization of RLM systems, requires further detailed studies. One can expect that this value depends on the interactions between atoms belonging to different molecules as well as on the ratio between diagonals of RLM molecules.

Interestingly, the presented herein results could be explained in term of the classical nucleation theory. The nucleation rate for the system with *μ* oriented perpendicularly the longest molecular axis is much higher than for system possessing *μ* of the same value but oriented parallel to the longest molecular axis. Consequently, the specific replacement of single atom within the molecule leading to the change of the dipole moment orientation could result in entirely different stability behavior of the system. Taking this fact into account, we can conclude that the molecular architecture, which is not considered in simulations of common model systems, has a crucial meaning for macroscopic properties of the system. At this point it must be noted that the structural anisotropy and the spatial charges distribution are not all components of the molecular architecture. Thus, the presented in this paper studies indicate on the completely unexplored direction for feature computational studies, which focus on the effect of given ingredient of molecular architecture in the structural, dynamic, and thermodynamic behaviors of real systems.
